# Has tumor doubling time in breast cancer changed over the past 80 years? A systematic review

**DOI:** 10.1002/cam4.3939

**Published:** 2021-07-15

**Authors:** Meryl Dahan, Delphine Hequet, Claire Bonneau, Xavier Paoletti, Roman Rouzier

**Affiliations:** ^1^ Department of Surgery Institut Curie Hospital Group Saint‐Cloud France; ^2^ Inserm U900 Cancer et génome: bioinformatique biostatistiques et épidémiologie Institut Curie Saint‐Cloud France; ^3^ University Versailles St‐Quentin University Paris‐Saclay Montigny‐le‐Bretonneux France

**Keywords:** breast cancer, molecular subtypes, screening, tumor doubling time, tumor growth rate

## Abstract

Over the past century, epidemiologic changes and implementation of screening may have had an impact on tumor doubling time in breast cancer. Our study was designed to evaluate changes in tumor doubling time in breast cancer over the past 80 years. A systematic review of published literature and meta‐regression analysis was performed. An online electronic database search was undertaken using the PubMed platform from inception until June 2020. All studies that measured tumor doubling time in breast cancer were included. A total of 151 publications were retrieved. Among them, 16 full‐text articles were included in the qualitative analysis. An exponential growth model was used for quantitative characterization of tumor growth rate. Tumor doubling time has remained stable over the past 80 years. Recent studies have not only identified “fast growing tumor” (grade 3, human epidermal growth factor receptor 2‐positive, triple‐negative, or tumor with an elevated Ki‐67) but also “inactive breast cancer” feeding the ongoing debate of overdiagnosis due to screening programs. The stability of tumor doubling time over the past 80 years, despite increasing and changing risk factors, supports the validity for our screening guidelines. Prospective studies based on more precise measurement of tumor size and adjustment for tumor characteristics are necessary to more clearly characterize the prognostic and predictive impact of tumor doubling time in breast cancer.

## Graphical abstract

The stability of tumor doubling time over the past 80 years, despite increasing and changing risk factors, supports the validity of our screening guidelines. Recent studies have not only identified “fast growing tumor” but also “inactive breast cancer” feeding the ongoing debate of overdiagnosis due to screening programs.

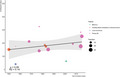

## 1 BACKGROUND

Tumor doubling time (DT) is defined by the number of days required for a tumor to double its volume. A shorter DT indicates a faster tumor growth rate.^1^ The duration of the cell cycle is similar in tumors and healthy tissues, but tumors present a higher proportion of cells undergoing mitosis. This proportion of cells is called: “the growth fraction”, which is highly variable and dependent on the type of tumor. Metastases commonly have a growth rate almost twice that of the primary tumor.^2^, ^3^ Tumor DT is an important element for cancer progression prediction models and depends on the duration of the cell cycle, the growth fraction, and the rate of cell loss.^4^ A better understanding of tumor growth dynamics is essential in order to plan and evaluate optimal screening programs.^5^ Breast cancer (BC) is the most common cancer in women worldwide. BC incidence is increasing, especially as a result of modifiable exposures (alcohol consumption, physical inactivity, exogenous hormones such as hormone replacement therapy, and obesity).^6^ However, early diagnosis and improved management have significantly increased survival of breast cancer patients.^2^ Optimal screening plays a major role in patient prognosis and has now been implemented in most developed countries. An evolution of DT in breast cancer over time would lead to a revision of screening interval. It may also have an impact on the follow‐up schedule and recommendation of delay before surgery. A better acknowledgment of tumor growth dynamic in breast cancer could guide surgeons in their surgical timelines. Wait times for breast cancer surgery have increased over the past decade.^7^ Waiting times could cause additional anxiety for breast cancer patient; improved knowledge will reassure patients while they wait.^8^

Furthermore, tumor growths patterns according to molecular subtypes is a current major focus, and only few recent studies analyze it in terms of DT.

This systematic review was designed to evaluate changes in the DT in breast cancer over the past 80 years in order to assess the impact of epidemiologic changes and implementation of screening on DT that currently remains unknown.

## 2 METHODS

This study was based on a systematic review and meta‐regression analysis of the published literature in accordance with PRISMA guidelines.^9^

### 2.1 PICo question

The population (or problem), interest, and context (PICo) question of this systematic review was as follows: “Has tumor doubling time in breast cancer changed over the past 80 years?”.

### 2.2 Inclusion and exclusion criteria

Inclusion criteria were as follows: all studies that measured DT in breast cancer or analyzed the factors that may affect tumor doubling time (tumor grade, molecular subtype, and Ki‐67) with no restriction concerning the type of study.

We excluded studies not published in English and experimental studies on animal models.

### 2.3 Data sources and searches

An online electronic database search was conducted using the PubMed platform and adapted for use with other databases (Medline and Web of science) according to their search system. Any publication from inception to June 2020 was considered for inclusion. We used the following combination of MESH terms in our systematic review: “breast cancer” OR “breast neoplasm” AND “doubling time” AND “growth rate”. We completed our search by manual review of other related articles identified during the search. We first excluded studies according to the relevance of their titles and their abstracts. Full‐text articles were assessed for eligibility. Publications were reviewed by two authors and a third reviewer was consulted in the case of disagreement.

### 2.4 Data extraction

We extracted the following data: authors, year of publication and inclusion, size of the patient population, tumor size at diagnosis, tumor stage at diagnosis (T), lymph node involvement, interval between two measurements, formula used to calculate tumor volume, the model used to calculate DT, and the tumor doubling time (DT). When available, we collected DT according to tumor histological subtype (triple‐negative (TN), human epidermal growth factor receptor 2‐positive (HER2+), and hormone receptor‐positive (HR+), and HER2‐ (luminal) breast cancers, grade, and Ki‐67).

To reduce missing data to a minimum, we contacted the various authors to retrieve unpublished data, reconstructed certain plots, and assigned adjustment weights to some variable according to sample size.

We considered it more appropriate to collect the mean date of inclusion for each study rather than the year of publication. For two studies,^10^, ^11^ we estimated the mean date of inclusion according to the mean interval between the mean date of inclusion and publication of the other 14 studies. For some studies, we converted median DT values into mean values using an exponential model formula (median = ln2/λ, mean = 1/λ). Lee et al. calculated the tumor growth rate by means of the specific growth rate (SGR) formula. For the homogeneity of the review, we converted SGR (%/day) into DT (days) using the following formula: DT = ln2/SGR.^12^

### 2.5 Statistical analysis

Univariable linear regression analysis adjusted for sample size was used to plot DT over time. A positive slope indicates a longer DT over time, while a negative slope indicates a shorter DT. Wald tests for this parameter were used to test for a statistically significant effect. For studies in which DT was reported by subgroups (HER2+, triple‐negative, or luminal), we considered each subgroup separately.

All analyses were performed with R software (http://cran.r‐project. Org). A p‐value <0.05 was considered to be significant.

### 2.6 Quality assessment

We used a quality assessment tool elaborated by Hawker et al. in 2002^13^ (Appendix 1). This tool was elaborated for systematic review of qualitative evidence. The scale contains nine items assessing abstract/title, introduction/aims, method/data, sampling, data analysis, ethics/bias, results, transferability, and implications. Each item can be answered by “good”, “fair”, “poor”, and “very poor”. Lorenc et al. added a graduation to this scale.^14^ They assigned numerical scores to the answers from 1 point (very poor) to 4 points (good) to provide a final score of each study (9 to 36 points). The overall quality grades were defined by the following description: grade A (high quality), 30–36 points; grade B (medium quality), 24–29 points; and grade C (low quality), 9–24 points.

In our study, we used the scale of Hawker et al. and cut‐off values updated by Lorenc et al.^13^, ^14^ Two investigators reviewed all articles included and independently provided a final score for each study. If they found differing scores, the discrepancy was resolved by discussion.

## 3 RESULTS

### 3.1 Study selection

**FIGURE 1:**
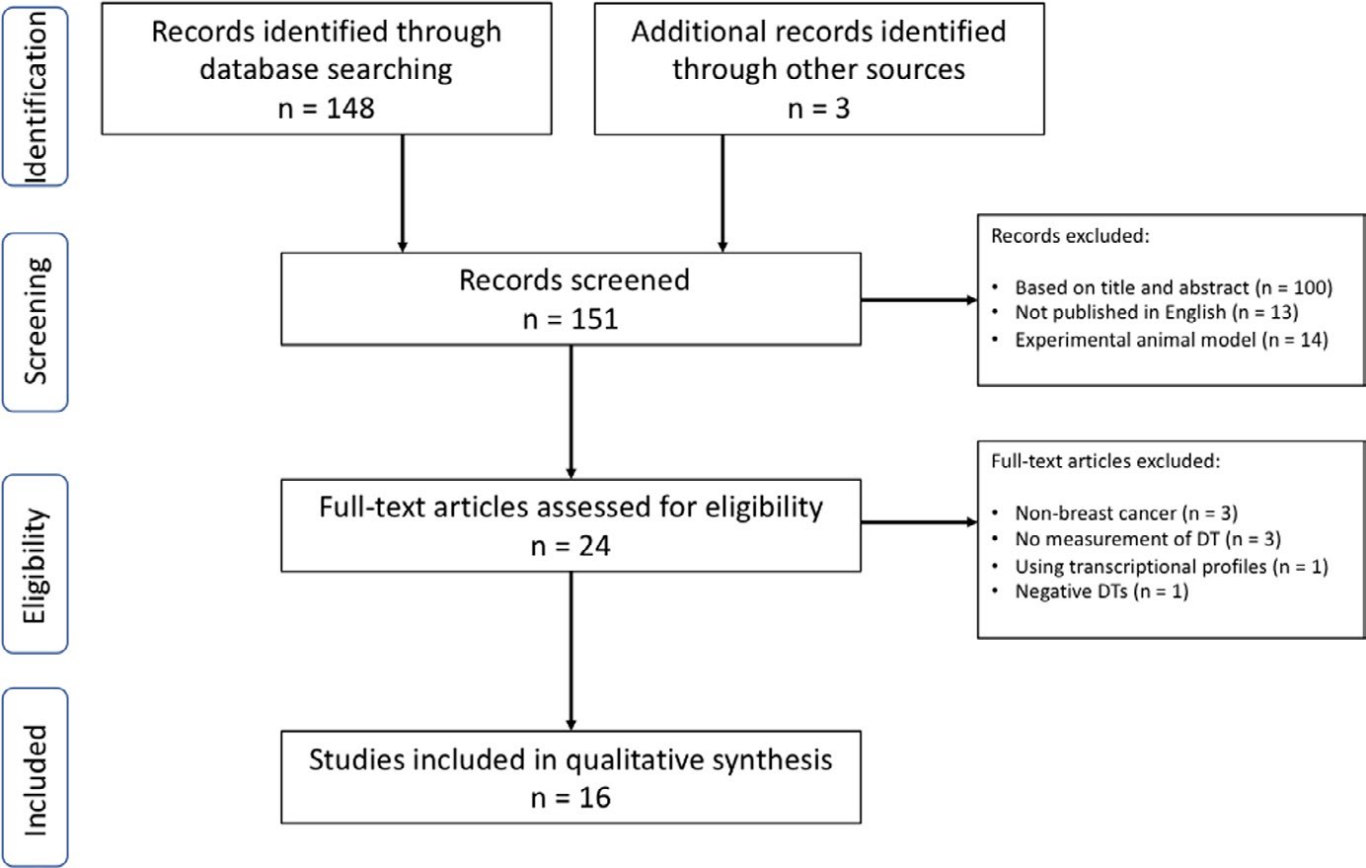
PRISMA Flow diagram for literature search

Our search produced 151 publications, including 3 additional records identified by sources other than PubMed. One hundred records were excluded after reviewing the title and abstract as they failed to meet the study inclusion criteria. Thirteen studies not published in English and 14 experimental studies were also excluded. Twenty‐four full‐text articles were assessed for eligibility. Seven studies were excluded because they failed to meet the inclusion criteria. One study was excluded because the authors included negative DT of tumors that had decreased in size without adjustment, leading to the shortest DT (15 days) reported in the literature, which was not comparable with the DT reported in other studies.^15^ Sixteen studies were, therefore, finally included in the qualitative analysis (Figure 1).

### 3.2 Tumor doubling time measurement methods and patient characteristics

Sixteen studies were included in our review and their results are summarized in Table 1. Tumor dimensions were measured by ultrasonography in 5 studies^4^, ^16^, ^17^, ^18^, ^19^ and by mammography in 10 studies^4^, ^10^, ^11^, ^20^, ^21^, ^22^, ^23^, ^24^, ^25^, ^26^ (Table 2). The mean time interval between two measurements varied considerably between studies, ranging from 8 days to 132 months (Table 2). Tumor volume was mainly calculated (in 11 studies) by the formula of a spheroid or the formula of a sphere: 4/3 π*abc* (a, b, and c were the 3 radii of the tumor) or 4/3 πr^3^ (where r was the largest diameter of the tumor), respectively. An exponential model was widely used to measure tumor growth rate. All but one of the publications used doubling time (days) for quantitative characterization of tumor growth rate. Lee et al. used specific growth rate (%/day), equal to ln2/DT, to quantify tumor growth rate.^17^ Patient characteristics are reported in Table 1. Twelve studies included non‐inflammatory primary breast cancer only. Four studies included T4 tumors, local recurrences, and distant metastasis.^4^, ^10^, ^27^, ^28^ T stage at diagnosis was mainly T1 or T2. The proportion of patients with lymph node involvement was greater than 50% in studies that enrolled patients before 1990, then significantly decreased over time on adjusted linear regression (*p *= 0.001). We did not find any correlation between the proportion of T1 and n0 tumors in the studies and DT (*p *= 0.79 and 0.59, respectively).

**TABLE 1:** Patient characteristics

Author Year of publication	Patient population	Age	Tumor diameter at diagnosis (mm)	T stage at diagnosis	Lymph node involvement
Gershon‐Cohen et al.^9^ 1963	n = 18	NA	18^a^ 20^b^	T1: 78% (n = 14) T2: 22% (n = 4) T3: 0% (n = 0) T4: 0% (n = 0)	Positive: 44% (n = 8) Negative: 56% (n = 10)
Philippe et al.^24^ 1968	n = 78 Local recurrences only	56^a,^, ^e^ <50: 22% (n = 17) ≥50: 76% (n = 59) NA: 2% (n = 2)	NA	NA	NA
Kusama et al.^23^ 1972	n = 199 Including primary BC, local metastasis, lymph node metastasis, pulmonary metastasis, and other metastasis	57^a^ <50: 33% (n = 66) ≥50: 67% (n = 133)	NA	NA	NA
Lundgren et al.^16^ 1977	n = 13	61^a^ <50: 23% (n = 3) ≥50: 77% (n = 10)	7.5^a^	T1: 92% (n = 12) T2: 8% (n = 1) T3: 0% (n = 0) T4: 0% (n = 0)	NA
Heuser et al.^17^ 1979	n = 32	NA	16.5^a^ 12.5^b^	T1: 78% (n = 25) T2: 19% (n = 6) T3: 3% (n = 1) T4: 0% (n = 0)	Positive: 81% (n = 26) Negative: 19% (n = 6)
Von Fournier et al.^18^ 1980	n = 147	61^a^ <50: 31% (n = 45) ≥50: 69% (n = 102)	17^a^ 18^b^	NA	Positive: 61% (n = 89) Negative: 24% (n = 36) NA: 15% (n = 22)
Galante et al.^19^ 1986	n = 196	NA	NA	NA	Positive: 50% (n = 98) Negative: 42% (n = 82) NA: 8% (n = 16)
Tabbane et al.^8^ 1989	n = 75 (n= 42 non‐advanced BC, n= 30 advanced T4 and/or N2 or N3, and n= 4 distant metastasis)	48^a^, ^e^ <50: 57% (n = 43) ≥50: 43% (n = 32)	NA	T0: 8% (n= 6) T1: 8% (n= 6) T2: 31% (n= 23) T3: 36% (n= 27) T4: 13% (n= 10) Tx: 4% (n= 3)	Positive: 84% (n = 63) Negative: 16% (n = 12)
Kuroishi et al.^4^ 1990	n = 122	43^a^, ^e^ <50: 67% (n = 82) ≥50: 25% (n = 31)	NA	T0: 1% (n=1) T1: 38% (n= 47) T2: 46% (n= 56) T3: 7% (n= 9) T4: 7% (n= 8) Tx: 1% (n= 1)	Positive: 57% (n = 70) Negative: 43% (n = 52)
Peer et al.^20^ 1993	n = 289	62^a^ <50: 16% (n = 46) ≥50: 84% (n = 243)	NA	NA	NA
Tilanus et al.^22^ 2005	n = 55 (n = 30 BRCA carriers, n = 25 BRCA non‐carriers)	42^a^, ^e^ BRCA: 40^a^ (Min 27‐ Max 52) Non‐BRCA: 45^a^ (Min 31–Max 59)	6^a^, ^f^4^a^, ^e^	T0‐T1: 74% (n = 41) T2: 4%(n = 2) T3: 0% (n = 0) T4: 0% (n = 0) NA: 22% (n = 12)	NA
Ryu et al.^12^ 2014	n = 66 ER+ (n = 37) HER2+ (n = 12) TN (n = 17)	50^a^ <50: 52% (n = 34) ≥50: 48% (n = 32) (Min 29–Max 78)	8.4^a^, ^e^ ER+: 7.6^a^ ±3.3 HER2+: 10.3^a^ ±7.3 TN: 8.9^a^ ± 5.1	NA	Positive: 11% (n = 7) Negative: 89% (n = 59)
Fornvik et al.^21^ 2015	n = 31	62^a^ ±12 (Min 42– Max 87)	19.5^a^ ±13.4 (Min 7– Max 80)	T1: 68% (n = 21) T2: 29% (n = 9) T3: 3% (n = 1) T4: 0% (n = 0)	Positive: 23% (n = 7) Negative: 74% (n = 23) NA: 3% (n = 1)
Lee et al.^13^ 2016	n = 323	53^a^ <50: 36% (n = 117) ≥50: 64% (n = 206) (Min 27– Max 82)	14.7 ^a^ ±6.1	T1: 81% (n = 262) >T1: 19% (n = 61)	Positive: 19% (n = 62) Negative: 81% (n = 261)
Zhang et al.^15^ 2017	n = 69	<52: 54% (n = 37) ≥52: 46% (n = 32)	12	NA	Positive: 14% (n = 10) Negative: 86% (n = 59)
Nakashima et al.^14^ 2018	n = 265	60.1 ± 12.2^a^	19.2^a^ ±10.9	T1: 64% (n = 170) T2: 34% (n = 89) T3: 2% (n = 6) T4: 0% (n = 0)	Positive: 31% (n = 83) Negative: 68% (n = 180) NA: 1% (n = 2)

^a^ Mean ± SD.

^b^ Median.

^c^ Median values were converted into mean values with the formula of an exponential model (median = ln2/λ, mean = 1/λ).

^d^ We converted SGR into DT with ln2/SGR formula.

^e^ We assigned adjustment weights to sample size.

^f^ We reconstructed plot.

**TABLE 2:** Tumor doubling time study endpoints

Author	Mean date of inclusion	Measurement method and interval (days)	DT (days) or SGR (%/days)
Gershon‐Cohen et al.^11^	1956	xR and surgery (Min 180–Max 1620)	DT: 175^a^, ^c^ 120^b^ (Min 23–209 Max)
Philippe et al.^28^	1956	NA	DT: 40^a^ (Min 3–Max 211)
Kusama et al.^27^	1950	NA	DT: 151^a^, ^c^ 105^b^ (Min 6–540 Max)
Lundgren et al.^20^	1972	xR 377^a^ (Min 95–Max 1950)	DT: 211^a^ (Min 42–397 Max)
Heuser et al.^21^	1977	xR (Min 88–Max 365)	DT: 325^a^ (Min 109–944 Max)
Von Fournier et al.^22^	1968	xR 810^a^ (Min 60–Max 3960)	DT: 212^a^ (Min 44–1869 Max)
Galante et al.^23^	1977	xR 30^a^	DT: 141^a^, ^e^ DT ≤ 30: 15.8% 30 < DT < 90: 42.9% DT ≥ 90: 41.3%
Tabbane et al.^10^	1982	xR or clinical 210^a^, ^e^ (Min 24–Max 1907)	DT: 186^a^ 115^b^ (Min 14–772 Max)
Kuroishi et al.^4^	1983	Clinical or US or xR (Min 15–Max 2730)	DT: 174^a^ (Min 11‐Max 1293)
Peer et al.^24^	1985	xR (Min 180–Max 1825)	DT: 151^a^, ^e^ Age < 50: 80^a^ (95%CI 44–147) Age 50–70: 157^a^ (95%CI 121–204) Age > 70: 188^a^ (95%CI 120–295) *p* = 0.04
Tilanus et al.^26^	2001	MRI (n = 21) xR (n = 34) 328^a^ (Min 109‐ Max 657)	DT: 6^a^, ^e^ Carriers: 45^a^ (CI 26–73) Non‐carriers: 84^a^ (CI 58–131) *p* = 0.048
Ryu et al.^16^	2007	US: 372^a^, ^e^ ER+: 391 ± 214^a^ HER2+: 393 ± 239^a^ TN: 316 ± 105^a^	DT: 193 ± 141^a^ 141^b^ (Min 46–Max 825)
Fornvik et al.^25^	2014	xR 837^a^	DT: 282 ± 167^a^ (Min 46–Max 749)
Lee et al.^17^	2014	US 32^a^ 31^b^ (Min 8–Max 78)	SGR: 0.396^a^ DT: 177^a,d^
Zhang et al.^19^	2014	US: 182 ± 81.9^a^	DT: 185^a^ ± 126 164^b^ (Min 66–Max 521)
Nakashima et al.^18^	2015	US: 56.9 ± 19.9^a^	DT: 251^a^, ^c^ 174^b^, ^e^ (IQR: 97–360)

Abbreviations: BC, breast cancer; CI, confidence intervalDT, doubling time (day);ER, estrogen receptor; HER2, human epidermal growth factor receptor 2; IQR, interquartile range; MRI, magnetic resonance imaging; SD, standard deviation; SGR, specific growth rate; TN, triple negative; US, ultrasonography; xR, mammography.

^a^ Mean ± SD.

^b^ Median.

^c^ Median values were converted into mean values with the formula of an exponential model (median = ln2/λ, mean = 1/λ).

^d^ We converted SGR into DT with ln2/SGR formula.

^e^ We assigned adjustment weights to sample size.

^f^ We reconstructed plot.

## 4 GROWTH RATE OVER TIME

**FIGURE 2:**
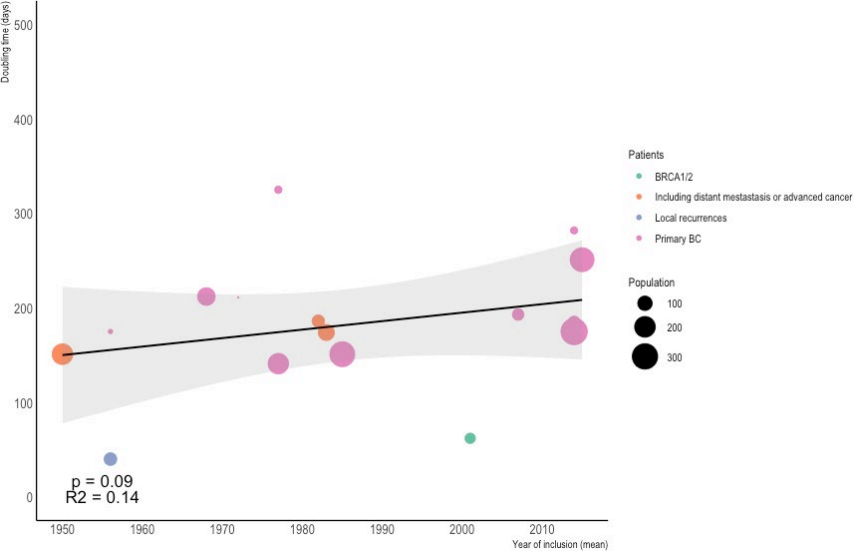
Linear regression analysis adjusted to sample size between mean DT and time

DT values are reported in Table 2. DT values have remained stable over the past 80 years. The linear equation adjusted for the study size had a slope of 1.03, which can be interpreted as an increase in the DT of 1.03 days per year (Figure 2). However, this time trend was not statistically significant (*p* = 0.09, R^2^ = 0.14).

## 5 HISTOPATHOLOGICAL EVALUATION

**FIGURE 3:**
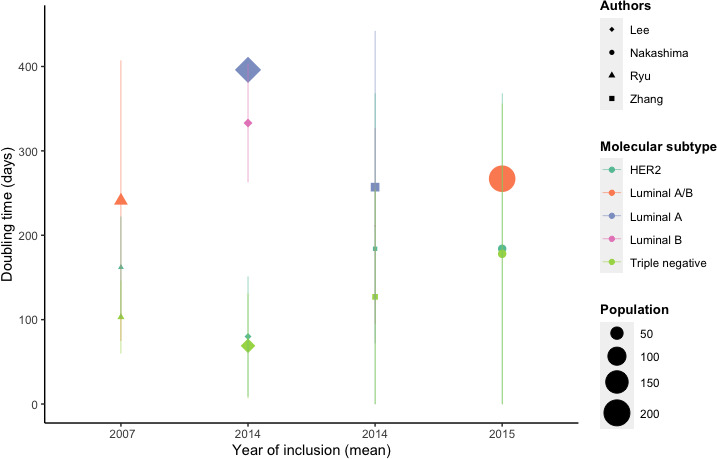
DT according to tumor molecular subtype (vertical lines are SD)

Six studies evaluated the impact of tumor characteristics on DT, and their results are summarized in Table 3. All four articles^16^, ^17^, ^18^, ^19^ that reported the impact of molecular subtypes on DT reported significantly shorter DTs for TN and HER2+ tumors compared to luminal breast cancers (Figure 3). In their study, Ryu et al. reported a DT of 103 ± 43 days for triple‐negative breast cancer and a DT of 162 ± 60 days for HER2+ breast cancer compared to a DT of 241 ± 166 days for ER+breast cancer (*p *< 0.0001).^16^ Zhang et al. also reported a DT of 127 ± 48 days for triple‐negative breast cancer, 184 ± 71 days for HER2+ breast cancer, and 257 ± 185 days for luminal A breast cancer (*p* = 0.013).^19^ Four studies^10^, ^17^, ^18^, ^25^ also reported a significantly shorter DT in grade 3 tumors compared to grade 1 or 2 tumors. Ryu et al. and Zhang et al. did not find any significant difference in DT according to tumor grade.^16^, ^19^ An elevated Ki‐67 index was significantly associated with shorter DT.^16^, ^17^, ^18^, ^19^ Ryu et al. reported a DT of 205 ± 146 days for tumors with a Ki‐67 index <14% compared to 114 ± 78 days for tumors with a Ki‐67 index ≥14% (*p* = 0.004).^16^

**TABLE 3:** Doubling time according to tumor characteristics

Author	Molecular subtypes		Histological grade				Ki−67 (%)		
Tabbane et al.^10^ n = 75		NA		Grade 1	DT<90: n = 1	*p* = 0.01	NA		
					DT 90–180: n = 1				
					DT>180: n = 8				
				Grade 2	DT<90: n = 8				
					DT 90–180: n = 8				
					DT>180: n = 10				
				Grade 3	DT<90: n = 16				
					DT 90–180: n = 10				
Ryu et al.^16^ n = 66	ER+ (n = 37, 56%)	DT: 241 ± 166^a^	*p* < 0.0001	Grade 1	DT: 204 ± 149^a^	*p* = 0.090	<14%	DT:	*p* = 0.004
				(n = 13, 20%)			(n = 56, 85%)	205 ± 146^a^	
	HER2+ (n = 12, 18%)	DT: 162 ± 60^a^		Grade 2	DT: 230 ± 179^a^				
				(n = 25, 38%)			≥14%	DT: 114 ± 78^a^	
							(n = 10, 15%)		
	TN (n = 17, 26%)	DT: 103 ± 43^a^		Grade 3	DT: 154 ± 0.80^a^				
				(n = 28, 42%)					
Fornvik et al.^25^ n = 31		NA		Grade 1	DT: 296^a^	*p* = 0.002	NA		
				(n = 8, 26%)	(Min 147–Max 531)				
				Grade 2	DT: 352^a^				
				(n = 16, 52%)	(Min 139–Max 749)				
				Grade 3	DT: 105^a^				
				(n = 7, 22%)	(Min 46–Max 157)				
Lee et al.^17^ n = 323	Luminal A (n = 204, 63%)	SGR: 0.175 ± 0.979^a^ DT: 396 ± 71^d^	*p *< 0.001	Grade 1	SGR: 0.118 ± 1.009^a^	*p *< 0.001	<14% (n = 250, 77%)	SGR: 0.251 ± 1.012^a^ DT: 276^d^ ± 68	*p *< 0.001
				(n = 39, 12%)	DT: 587^d^ ± 69				
	Luminal B (n = 30, 9%)	SGR: 0.208 ± 0.996^a^ DT: 333 ± 70^d^		Grade 2	*SGR*: 0.183 ± 0.979^a^				
				(n = 155, 48%)	DT: 379^d^ ± 71				
	HER2+ (n = 22, 7%)	SGR: 0.859 ± 0.978^a^ DT: 80 ± 71^d^		Grade 3 (n = 129, 40%)	*SGR*: 0.736 ± 1.103^a^ DT: 94^d^ ± 63		≥14% (n = 73, 23%)	SGR: 0.892 ± 1.110^a^ DT: 78 ± 62^d^	
	TN (n = 67, 21%)	SGR: 1.003 ± 1.121^a^ DT: 69 ± 62^d^							
Zhang et al.^19^ n = 69	Luminal A (n = 29, 42%)	DT: 257 ± 185^a^	*p* = 0.013	Grade 1 (n = 15, 22%)	DT: 225^a^ ± 143	*p* = 0.116	<14% (n = 33, 48%)	DT: 224 ± 136^a^	*p* = 0.018
	Luminal B (n = 12, 17%)	DT: 211 ± 116^a^		Grade 2 (n = 42, 61%)	DT: 201^a^ ± 156		≥14% (n = 36, 52%)	DT: 145 ± 87^a^	
	HER2+ (n = 10, 15%)	DT: 184 ± 71^a^		Grade 3 (n = 12, 17%)	DT: 169^a^ ± 90				
	TN	DT: 127 ± 48^a^							
	(n = 18, 26%)								
Nakashima et al.^18^ n = 265	ER+/HER2‐ (n = 209, 79%)	DT: 267 ± 267^a^, ^c^ 185^b^ (IQR: 111–398)	*p* = 0.035	Grade 1	DT<90: (n = 4, 1%) DT>90: (n = 93, 35%)	*p *< 0.001	DT<90: 33^b^ (Min 15– Max 60)	DT>90: 16^b^ (Min 10–Max 31)	*p = *0.001
	ER+/HER2+ (n = 15, 6%)	DT: 238 ± 238^a^, ^c^ 165.3^b^ (IQR: 125–333)		Grade 2	DT<90: (n = 19, 7%) DT>90: (n = 95, 36%)				
	ER‐/HER2+ (n = 13, 5%)	DT: 123 ± 123^a^, ^c^ 85.4^b^ (IQR: 77–354)		Grade 3	DT<90: (n = 15, 6%) DT>90: (n = 39, 15%)				
	TN (n = 28, 10%)	DT: 178 ± 178^a^, ^c^ 123.6^b^ (IQR: 77–177)							

Abbreviations: BC, breast cancer; CI, confidence intervalDT, doubling time (day);ER, estrogen receptor; HER2, human epidermal growth factor receptor 2; IQR, interquartile range; MRI, magnetic resonance imaging; SD, standard deviation; SGR, specific growth rate; TN, triple negative; US, ultrasonography; xR, mammography.

^a^ Mean ± SD.

^b^ Median.

^c^ Median values were converted into mean values with the formula of an exponential model (median = ln2/ λ, mean = 1/λ).

^d^ We converted SGR into DT with ln2/SGR formula.

^e^ We assigned adjustment weights to sample size.

^f^ We reconstructed plot.

## 6 STUDY QUALITY

Results of the quality assessment are described in Table 4. Six studies were classified high quality (Grade A),^16^, ^17^, ^18^, ^19^, ^25^, ^26^ 5 studies were classified medium quality (Grade B),^4^, ^10^, ^22^, ^23^, ^24^ and the 5 earliest studies were of low quality (Grade C).^11^, ^20^, ^21^, ^27^, ^28^ Before the 2000 s, ethical issues were not raised. Moreover, authors did not critically examine their potential bias and limitations. After the 2000 s, studies had higher‐quality classification score. Methods were more specific, clearly described, and easier to understand. The description of statistical analysis was rigorous and discussed. Sample size was justified and findings were explicit and represented with tables and figures.

**TABLE 4:** Quality assessment

Author	Abstract/Title	Introduction/Aims	Method/Data	Sampling	Data analysis	Ethics/Bias	Results	Transferability	Implications	Total/Grade
Gershon‐Cohen et al.^11^	Very poor	Fair	Poor	Poor	Poor	Very poor	Fair	Poor	Fair	19/C
Philippe et al.^28^	Fair	Poor	Fair	Poor	Fair	Very poor	Fair	Poor	Fair	22/C
Kusama et al.^27^	Fair	Poor	Poor	Poor	Poor	Very poor	Fair	Poor	Poor	19/C
Lundgren et al.^20^	Poor	Fair	Fair	Poor	Poor	Poor	Fair	Very poor	Poor	20/C
Heuser et al.^21^	Fair	Very poor	Good	Fair	Fair	Very poor	Fair	Fair	Poor	23/C
Von Fournier et al.^22^	Good	Fair	Fair	Fair	Fair	Poor	Good	Fair	Fair	28/B
Galante et al.^23^	Good	Fair	Good	Poor	Poor	Poor	Fair	Poor	Fair	25/B
Tabbane et al.^10^	Fair	Fair	Fair	Fair	Poor	Very poor	Good	Fair	Fair	25/B
Kuroishi et al.^4^	Good	Fair	Good	Fair	Fair	Very poor	Good	Fair	Fair	29/B
Peer et al.^24^	Good	Fair	Good	Poor	Fair	Very poor	Fair	Poor	Fair	25/B
Tilanus et al.^26^	Good	Good	Good	Fair	Good	Very poor	Good	Good	Fair	31/A
Ryu et al.^16^	Good	Good	Good	Good	Good	Good	Good	Good	Good	36/A
Fornvik et al.^25^	Good	Good	Good	Good	Good	Good	Good	Good	Fair	35/A
Lee et al.^17^	Good	Good	Good	Good	Good	Good	Good	Good	Good	36/A
Zhang et al.^19^	Good	Fair	Good	Good	Good	Good	Good	Good	Good	35/A
Nakashima et al.^18^	Good	Good	Good	Good	Good	Good	Good	Good	Good	36/A

## 7 DISCUSSION

This review was designed to evaluate changes over time in the DT in breast cancer. To our knowledge, this is the first systematic review and meta‐regression analysis of tumor doubling time in breast cancer. In the 16 studies included in the qualitative analysis, the DT remained stable over the last 80 years, with an average of 180 days, suggesting that contemporary risk factors for breast cancer have increased the incidence of breast cancer more than the tumor growth rate. However, recent studies assessing the impact of tumor characteristics on DT have highlighted the existence of “inactive breast cancer” and “fast growing tumors”.^10^, ^16^, ^18^, ^19^, ^25^

A better knowledge of the DT can be useful to design optimal screening and follow‐up programs. Breast cancer screening programs are currently based on guidelines published at the end of the 1980 s.^29^ The interval between two mammograms may need to be revised since publication of these guideline, especially if the DT has changed over time. However, this review shows that the DT has remained stable over recent decades, indicating that our screening guidelines remain valid. Nakashima et al. and Heuser et al. found that 36% and 28% of tumors, respectively, did not increase in size on the second measurement and described these tumors as being “inactive”.^18^, ^21^ This result contributes to the ongoing debate concerning the risks and benefits of breast cancer screening, particularly the risk of overdiagnosis and overtreatment of patients with “inactive” breast cancer, which would never become clinically apparent during the patient's lifetime.^30^

The incidence of breast cancer has increased over recent decades, mainly as a result of modifiable exposures (obesity, exogenous hormones, alcohol consumption, etc.). Exposure to these risk factors may also have had an impact on the DT. None of the studies reviewed here included risk factors in their analysis. However, the stability of DT over the past 80 years suggests that modifiable exposures do not have any significant impact on DT in breast cancer. The histopathologic classification of breast cancer has become a major factor to guide the clinical management of breast cancer patients. Triple‐negative and HER2+ tumors have a poorer prognosis than luminal breast cancer and are usually treated by chemotherapy. Not surprisingly, these tumors have a short DT, which is consistent with their poor prognosis. However, it is unknown whether DT has a predictive value for chemosensitivity. It would be particularly useful to determine whether evaluation of DT between diagnosis and treatment initiation could constitute a prognostic factor. Similarly, with the growing number of window of opportunity (WOO) studies (trials in which patients receive one or more new compounds between their cancer diagnosis and standard treatment) in the field of breast cancer research, tumor growth dynamics must first be clearly elucidated. “Inactive” breast tumors could constitute a confounding factor in these studies.

We acknowledge that this study presents a number of limitations. One of the limitations of a meta‐analysis of observational studies is that no appropriate tools are available to assess publication bias. The best strategy to assess publication bias in observational studies in epidemiology is a thorough search, which was performed. One of the studies was prospective,^23^ while the other 15 studies were retrospective, mostly based on small sample sizes. Measurement intervals were highly variable and poorly defined in some studies. Different methods with several radiologists' perception were used to measure tumor size leading to potential measurement bias. The most recent studies considered ultrasonography (US) to be more appropriate than mammography to evaluate tumor volume.^31^ Several published studies concluded that magnetic resonance imaging (MRI) is the most appropriate examination for tumor size estimation.^32^, ^33^, ^34^ In order to improve DT calculation, future studies could use MRI to measure tumor size. The growing role of neoadjuvant chemotherapy could have led to selection bias especially in recent studies. Thus, triple‐negative, HER2+, or locally advanced cancers were most of the time excluded or less prevalent in recent studies.

Finally, the various studies included different patient populations. Studies including local recurrence, T4 stage, BRCA1/2 (breast cancer 1/2) mutation, or *de novo* distant metastasis could have influenced DT^4^, ^10^, ^26^, ^27^, ^28^ (Figure 2). Two main patterns of growth of human cancers are described in the literature: exponential and Gompertzian.^3^ In oncology, the Gompertzian model has been considered to be the best mathematical approach to tumor growth.^35^, ^36^, ^37^ However, the exponential model was most commonly used to model cancer progression in selected studies. This method is widely used because of the short measurement intervals for estimations of the volume of early untreated breast tumors.^12^, ^18^ In our review, an exponential model was often used to calculate the DT and a spheroid or sphere formula was used to estimate tumor volume, ensuring better comparability of studies in our study.

Lastly, our quality assessment highlighted a methodological and ethical measure improvement over the last 80 years. Concerns about ethical issues are potentially responsible for a decline in breast cancer natural history studies over time. Indeed, prospective studies analyzing tumor growth rate and potentially delaying therapeutic management would lead to inevitable ethical concerns. We believe that the biases and strengths identified in previous studies are important for the design of future high‐quality studies evaluating tumor doubling time in breast cancer.

## 8 CONCLUSION

The DT has not varied significantly over the past 80 years. Despite a qualitative improvement over the years, additional prospective studies based on larger sample sizes, more precise measurement of tumor size adjusted for risk factors, and tumor characteristics are necessary to more accurately characterize DT in breast cancer.

## CONFLICTS OF INTEREST

The authors declare that they have no competing interests.

## Authors’ contributions

MD, RR and DH conceived and designed the analysis. MD collected the data. MD, RR and XP analyzed the data. MD, DH, CB, RR and XP were involved in the drafting and critical review. MD and RR reviewed the articles for quality assessment. All authors read and approved the final manuscript.

## Ethics statement

Not applicable.

## Consent to participate/Consent to publish

Not applicable.

## Code availability

R software: http://cran.r‐project. Org

## Registration

The systematic review was registered on OSF (https://osf.io/jkvs4/).

## Data Availability

The datasets used and analyzed during the current study are available from the corresponding author on reasonable request.

## APPENDIX 1

### Quality assessment tool by Hawker et al.

1. **Abstract and title:** Did they provide a clear description of the study?
  - Good: Structured abstract with full information and clear title.
  - Fair: Abstract with most of the information.
  - Poor: Inadequate abstract.
  - Very Poor: No abstract.
2. **Introduction and aims**: Was there a good background and clear statement of the aims of the research?
  - Good: Full but concise background to discussion/study containing up‐to‐date literature review and highlighting gaps in knowledge. Clear statement of aim AND objectives including research questions.
  - Fair: Some background and literature review. Research questions outlined.
  - Poor: Some background but no aim/objectives/questions, OR Aims/objectives but inadequate background.
  - Very Poor: No mention of aims/objectives. No background or literature review.
3. **Method and data:** Is the method appropriate and clearly explained?
  - Good: Method is appropriate and described clearly (e.g., questionnaires included). Clear details of the data collection and recording.
  - Fair: Method appropriate, description could be better. Data described.
  - Poor: Questionable whether method is appropriate. Method described inadequately. Little description of data.
  - Very Poor: No mention of method, AND/OR Method inappropriate, AND/OR No details of data.
4. **Sampling:** Was the sampling strategy appropriate to address the aims?
  - Good: Details (age/gender/race/context) of who was studied and how they were recruited. Why this group was targeted. The sample size was justified for the study. Response rates shown and explained.
  - Fair: Sample size justified. Most information given, but some missing.
  - Poor: Sampling mentioned but few descriptive details.
  - Very Poor: No details of sample.
5. **Data analysis:** Was the description of the data analysis sufficiently rigorous?
  - Good: Clear description of how analysis was done. Qualitative studies: Description of how themes derived/respondent validation or triangulation. Quantitative studies: Reasons for tests selected hypothesis driven/numbers add up/statistical significance discussed.
  - Fair: Qualitative: Descriptive discussion of analysis. Quantitative.
  - Poor: Minimal details about analysis.
  - Very Poor: No discussion of analysis.
6. **Ethics and bias:** Have ethical issues been addressed, and what has necessary ethical approval gained? Has the relationship between researchers and participants been adequately considered?
  - Good: Ethics: Where necessary issues of confidentiality, sensitivity, and consent were addressed. Bias: Researcher was reflexive and/or aware of own bias.
  - Fair: Lip service was paid to above (i.e., these issues were acknowledged).
  - Poor: Brief mention of issues.
  - Very Poor: No mention of issues.
7. **Results:** Is there a clear statement of the findings?
  - Good: Findings explicit, easy to understand, and in logical progression. Tables, if present, are explained in text. Results relate directly to aims. Sufficient data are presented to support findings.
  - Fair: Findings mentioned but more explanation could be given. Data presented relate directly to results.
  - Poor: Findings presented haphazardly, not explained, and do not progress logically from results.
  - Very Poor: Findings not mentioned or do not relate to aims.
8. **Transferability or generalizability:** Are the findings of this study transferable (generalizable) to a wider population?
  - Good: Context and setting of the study is described sufficiently to allow comparison with other contexts and settings, plus high score in Question 4 (sampling).
  - Fair: Some context and setting described, but more needed to replicate or compare the study with others, PLUS fair score or higher in Question 4.
  - Poor: Minimal description of context/setting.
  - Very Poor: No description of context/setting.
9. **Implications and usefulness:** How important are these findings to policy and practice?
  - Good: Contributes something new and/or different in terms of understanding/insight or perspective. Suggests ideas for further research. Suggests implications for policy and/or practice.
  - Fair: Two of the above (state what is missing in comments).
  - Poor: Only one of the above.
  - Very Poor: None of the above.
